# Supplementary material for: Plant-Based Ingredients Utilized as Fat Replacers and Natural Antimicrobial Agents in Beef Burgers
Source: Foods. 2024 Oct 11;13(20):3229. doi: 10.3390/foods13203229 (PMC11507565; doi:10.3390/foods13203229)
Supplement: Supplementary file 1 [file foods-13-03229-s001.zip › foods-3214292-supplementary.pdf]

Table S1

Descriptive statistical analysis related to the sensory evaluation of different batches of burgers.

Attributes	Batches	Mean	Median	Min	Max	sd	(p < 0.05)
	C	7.6	7	5	10	1.5	a
**Appearance**	CBG	5.5	6	3	7	1.5	b
	A	7.8	8	6	9	0.9	a
	C	5.8	6	3	8	1.9	a
**Flavour**	CBG	6.4	7	3	9	1.7	a
	A	7.3	7	6	8	0.7	a
	C	4.9	4	3	8	1.8	a
**Juiciness**	CBG	6.5	7	4	9	1.6	b
	A	8.0	8	7	9	0.7	c
	C	5.9	6	4	8	1.0	a
**Residue**	CBG	7.0	7	5	8	1.1	b
	A	3.4	3	1	7	1.7	c
	C	5.9	6	3	9	1.9	a
**Taste**	CBG	6.5	7	3	9	1.8	a
	A	8.0	8	7	9	0.7	b
	C	5.6	5	3	9	1.8	a
**Tenderness**	CBG	6.6	7	4	9	1.3	ab
	A	7.7	8	6	9	1.0	b
	C	6.5	7	3	9	2.0	ab
**Visible fat**	CBG	5.8	6	3	8	1.7	a
	A	7.9	8	7	9	0.7	b

Different lowercase letters indicate significant ($p < 0.05$) differences between batches for each attribute.

Table S2

Antimicrobial action (inhibitory halo expressed by natural antimicrobial agents and tetracycline)

**Antimicrobial agents**	***L. innocua***	***P. fragi***	***P. putida***
**Nettle**	1.53 ± 0.06	1.85 ± 0.11	2.10 ± 0.12
**Medlar**	2.26 ± 0.08	2.46 ± 0.12	2.74 ± 0.10
**Tetracycline disk**	2.81 ± 0.09	2.83 ± 0.10	2.84 ± 0.10
